# Modified Smith–Petersen approach with rectus-sparing reduces severe avascular necrosis for developmental dysplasia of the hip at walking age: minimum 5-year follow-up

**DOI:** 10.1186/s13018-022-03441-6

**Published:** 2022-12-13

**Authors:** Mingyuan Miao, Sheng Jin, Haiqing Cai, Haoqi Cai, Jingxia Bian, Zhigang Wang

**Affiliations:** Department of Orthopedic Surgery, Shanghai Children’s Medical Center, School of Medicine, Shanghai Jiao Tong University, Shanghai, China

**Keywords:** Developmental dysplasia of the hip, Walking age, Smith–Petersen approach, Avascular necrosis

## Abstract

**Background:**

Developmental dysplasia of the hip (DDH) is one of the most common orthopedic malformations in children. Open reduction for DDH at walking age remains a major concern. The goal of this study is to evaluate the mid-term effect of a modified Smith–Petersen approach which preserves the rectus femoris on DDH at walking age, in particular avascular necrosis (AVN).

**Methods:**

A retrospective review of DDH patients aged between 12 and 24 months was carried out between January 2010 and June 2016. Open reduction through the Smith–Petersen approach (Group A) and modified Smith–Petersen approach, which preserves the rectus femoris (Group B), were both used. Measurement of hip geometry included acetabular index, the International Hip Dysplasia Institute classification, and AVN degree. Clinical records included operation time, bleeding volume, and abduction angle.

**Results:**

There were 101 children (119 hips) with DDH who met the inclusion criteria. There were 66 hips in Group A and 53 in Group B. The mean surgical age at open reduction was 17.0 ± 2.4 months, with a mean 104.9 ± 19.5 months at last follow-up. There was no statistical difference in surgical age between the two groups (17.2 vs. 16.4 months). There was no significant difference in the incidence of all types of clinically significant AVN between group A and group B (27.3 vs. 18.9%), but the incidence of severe AVN was lower in group B (19.7 vs. 5.7%, *P* = 0.026). In addition, the lower the age at the time of open reduction, the lower the severity of AVN (*P* = 0.002).

**Conclusions:**

These mid-term data suggest that the modified Smith–Petersen approach with rectus-sparing could reduce severe AVN more than the classical Smith–Peterson approach in open reduction in DDH at walking age. In addition, early open reduction can reduce the postoperative degree of AVN.

## Introduction

DDH is one of the most common orthopedic malformations in children. The term DDH refers to the complete spectrum of abnormalities involving the growing hip, with varied expression from dysplasia to subluxation to dislocation of the hip joint [1]. At present, few cities in China offer routine ultrasound screening of hip joints for infants with suspected DDH. Upright walking can put the dislocated hip under abnormal stress, thus aggravating the pathological changes of the hip joint in both the soft tissues and osseous architecture. Diagnosis of many DDH patients is often delayed until they start walking; therefore, the success rate of brace and closed reduction is significantly reduced. Although there is no consensus about DDH in infants of walking age, most pediatric orthopedists prefer open reduction, regardless of which surgical approach is taken [2].

The medial (Ludloff) and anterior (Smith–Petersen) approach are most commonly used in open reduction for DDH in walking age. The medial femoral circumflex artery traverses the hip anteromedial capsule and may be iatrogenically injured during capsulotomy in the medial approach [3]. In addition, open reduction through the medial approach cannot simultaneously facilitate capsulorrhaphy and is generally recommended before infants start walking [4]. The anterior approach directly approaches the contracted hip capsule and can remove intra-articular obstacles [5]. Due to the clear exposure of the hip joint capsule in the anterior approach, many scholars have tried to optimize this approach by improving surgical procedures. The Smith–Petersen and modified Smith–Petersen approach, which preserves the rectus femoris, have both been used in our hospital. Recent studies have evaluated a rectus-sparing approach in hip surgery with better flexion and extension [6, 7]. The modified Smith–Petersen approach preserving the rectus femoris is an encouraging and safe option for treating a teratologically dislocated hip [8]. However, the efficacy and complications of the modified Smith–Petersen approach in preserving the rectus femoris in developmental dislocation of the hip have rarely been reported.

The purpose of the present study was to determine whether preserving the rectus femoris tendon attachment in the Smith–Petersen approach would (1) reduce intraoperative blood loss and operation time and (2) decrease the rate of complications, especially AVN.

## Materials and method

**Fig. 1:**
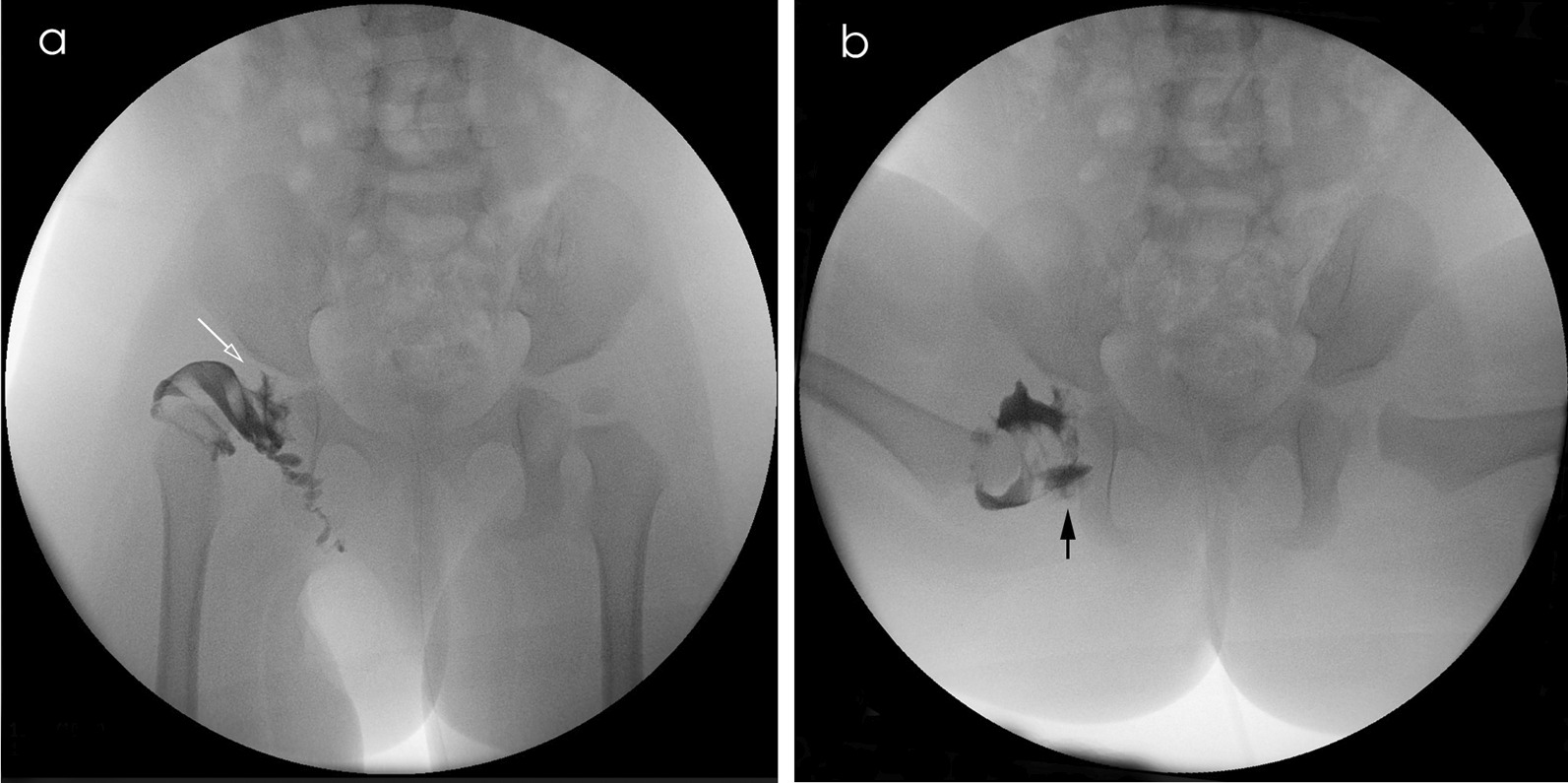
**a** Preoperative arthrography of right hip in a 16-month-old girl shows the hip inverted limbus (the white hollow arrow). **b** Tentative closed reduction shows that the right dislocated hip was irreducible, with widened acetabulum medial pooling (the black solid arrow)

Following ethical approval from the institutional review board at our hospital, we reviewed the clinical and radiological records of all children with DDH aged between 12 and 24 months at the time of surgery and who underwent surgery using a classical Smith–Petersen approach or modified Smith–Petersen approach between January 2011 and February 2022 (Fig. 1).

### Inclusion criteria

Patients were included if (1) they were 12 to 24 months of age at open reduction, after an initial attempt at closed reduction failed (2) using a Smith–Petersen approach, with or without rectus-sparing, and (3) were at a minimum of 5-year postoperative follow-up.

### Exclusion criteria

Patients were excluded if (1) they were below 12 months or over 24 months of age at open reduction, (2) had a teratogenic or neuromuscular dislocation, and (3) were at less than 5-year follow-up.

### Surgical technique

**Fig. 2:**
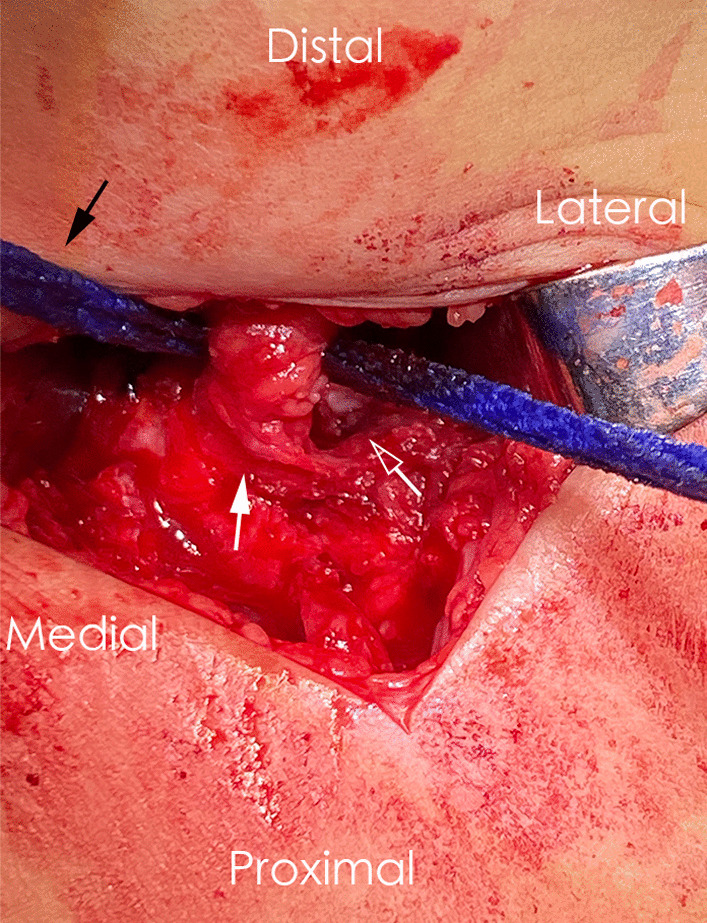
Photographs during right hip operation on a 15-month-old girl indicated the proximal rectus femoris was not released (the white solid arrow) in the modified Smith–Petersen approach and retracted by a sterile rope (the black solid arrow). The anterolateral femoral head (the white hollow arrow) was exposed after the hip joint capsule was opened through a T-shaped incision

Intraoperative dynamic arthrography with fluoroscopy was conducted to assess the quality of the closed hip reduction [9]. We used a medial approach (subadductor) for hip arthrography. Lohexol (300 mg/ml) was diluted with sterile saline at a ratio of 2:1, and a single hip received 0.5–1.5 ml of diluent. We used a 20-gauge intravenous catheter needle connected to a 5-ml syringe with a disposable extension tube to offer a sufficient reservoir for the dye. If the quality of closed reduction is poor and open reduction should be attempted, the surgeon uses the Smith–Petersen or modified Smith–Petersen approach through an incision below the iliac crest. In addition to percutaneous adductor tenotomy, the iliac apophysis is split, and then, the iliac wing is exposed subperiosteally. Whether the reflected and straight heads of the rectus femoris are released depends on the surgeon’s habits and intraoperative judgment. If the rectus femoris is not amputated during the operation (Fig. 2), the proximal rectus femoris in the surgical field could be pulled away by a hook or rope. The proximal rectus femoris is retracted laterally or medially to expose the anterolateral or posterolateral part of the hip capsule. The iliopsoas tendon is divided close to its attachment to the lesser trochanter of the femur. The hip capsule is opened parallel to its attachment, extending well into the medial false cavity.

The cavity of the acetabulum is cleared of any hindering soft tissue, including the ligamentum teres or its remnants. The transverse ligament and inferior capsule are also divided. Finally, the femoral head is gently reduced into the acetabulum without tension and, subsequently, hip capsulorrhaphy is performed by translationally tightening sutures with flexion and abduction. Then, the incision is closed layer by layer.

**Fig. 3:**
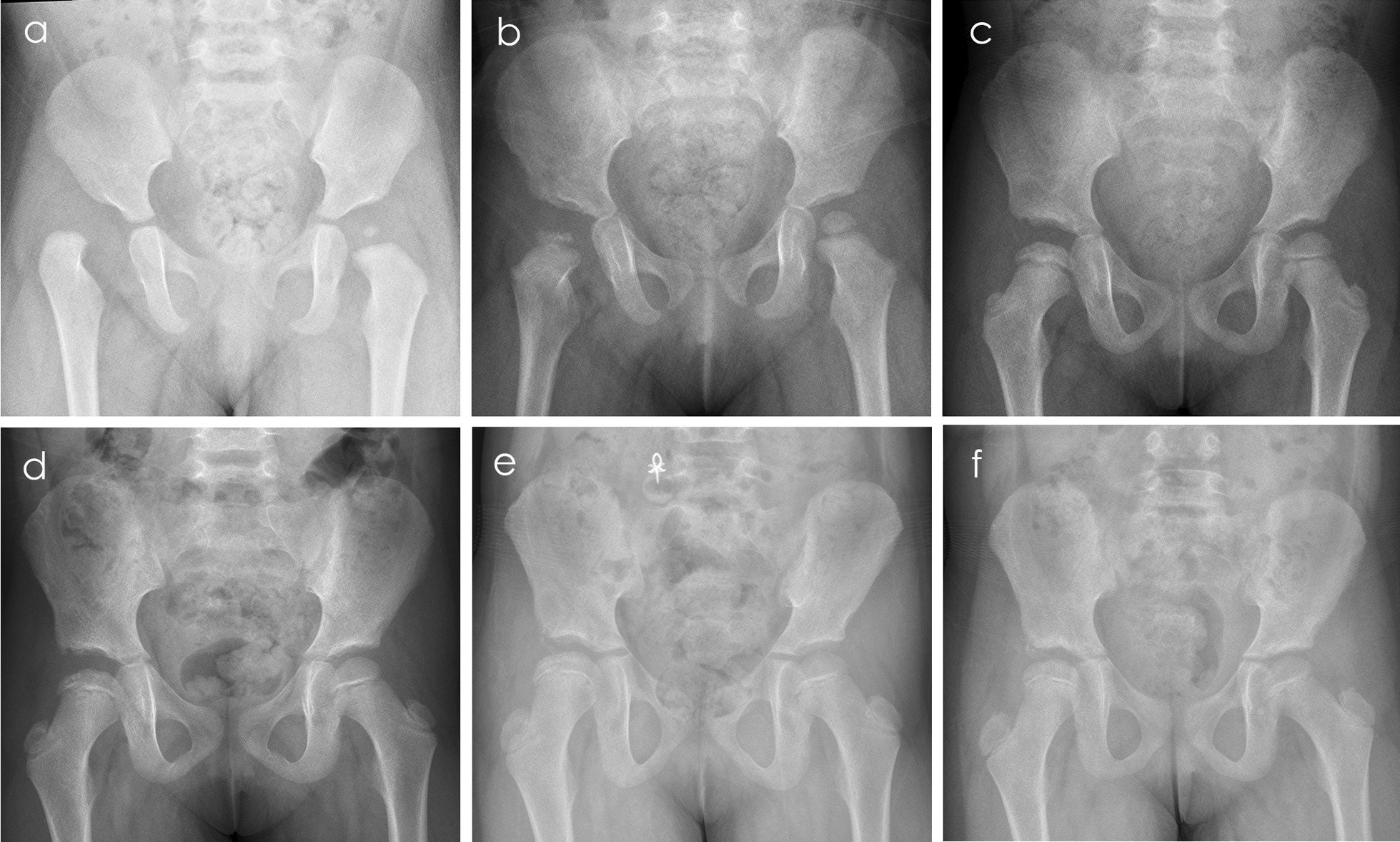
**a** Preoperative pelvic anteroposterior view in a 15-month-old girl on right hip dislocation with the classical Smith–Petersen approach. **b–f** Radiograph at 10, 40, 54, 75, and 87 months after open reduction, respectively, which shows Type 2 AVN

After the procedure above is carried out, the hips are maintained in a spica cast in human position with flexion and abduction. The results are confirmed with an immediate postoperative CT scan (Fig. 3). The fixation time is 12 weeks in spica cast; after that, an abduction brace is used for another four to six months.

Patients who underwent the classical Smith–Petersen approach were classified as group A, and those with rectus-sparing were classified as Group B.

The amount of bleeding and operation time was recorded. The operation time was defined as the time from skin cutting to skin closing.

The requirement for further surgical intervention, including pelvic osteotomy and femoral osteotomy, was noted from the records.

### Abduction angle in cast

The hip abduction angle in the spica cast was the complementary angle between the axis of the femoral shaft and the horizontal line tangent to bilateral ischial tuberosity through CT on cross section. These complementary angle values were expressed in degrees [10].

### Radiological outcome

**Fig. 4:**
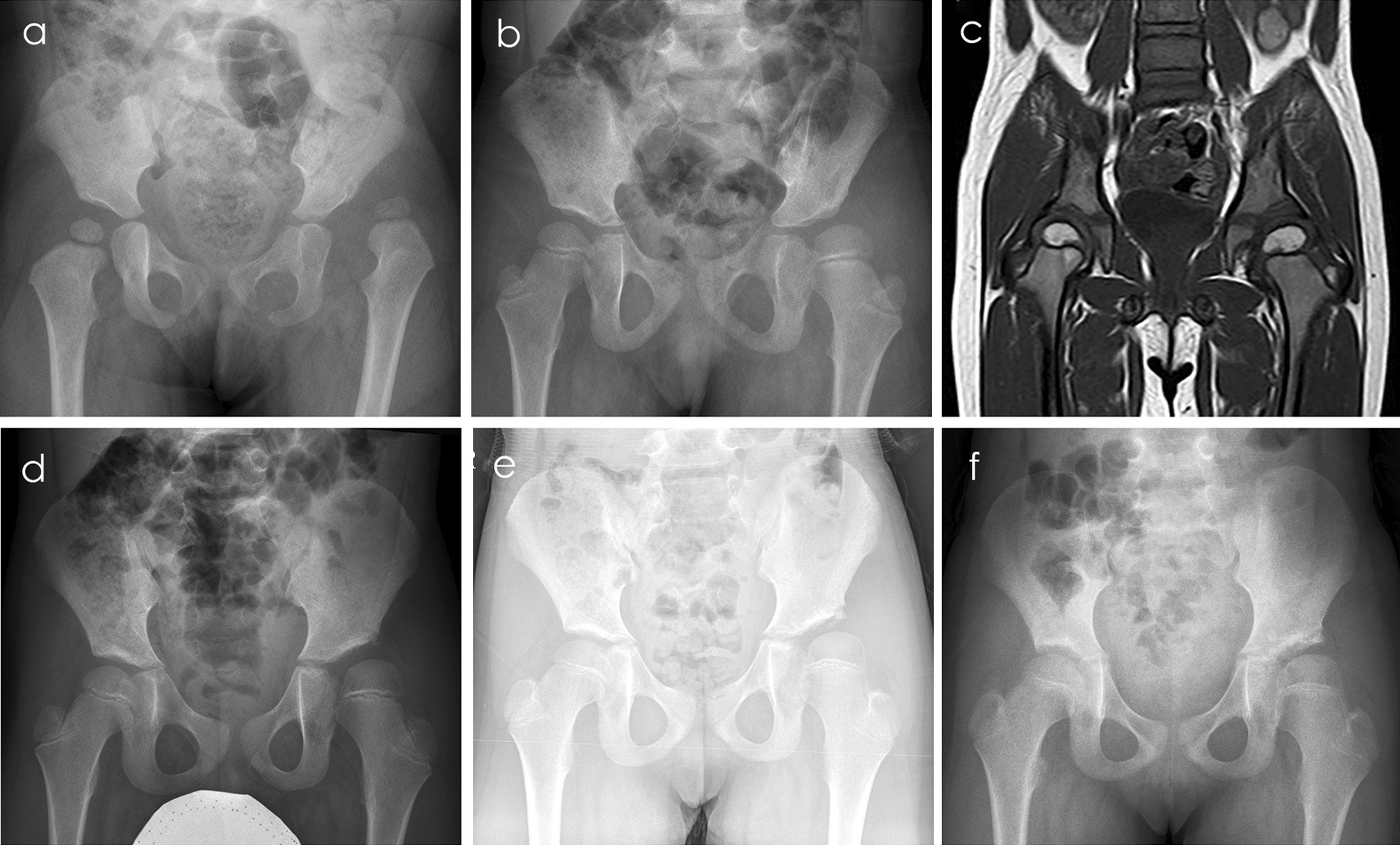
**a** Preoperative pelvic anteroposterior view in a 17-month-old girl on left hip dislocation with the modified Smith–Petersen approach. **b, d–f** Radiograph at 32, 64, 90, and 103 months after open reduction, respectively, without obvious AVN sign. **f** demonstrates suspicious early signs of type 2 AVN such as slightly laterally inclined proximally convex physis, medial femoral head flattening and an oval-shaped epiphysis. **c** MRI assessment at 32 months after open reduction

The following were noted on a preoperative anteroposterior (AP) pelvic radiograph: the IHDI classification, the presence of an ossific nucleus, and the acetabular index (AI). The Kalamchi and MacEwen classification was used to assess AVN at 2 and 5 years postoperatively and at the latest follow-up (Fig. 4). All classifications were determined by consensus among three authors (Mingyuan Miao, Jingxia Bian, and Zhigang Wang).

### AVN reporting

Radiological AVN outcomes were assessed according to the Kalamchi and MacEwen classification [11]. Type 1 AVN is identical in the Kalamchi and MacEwen classifications but does not result in serious complications and was therefore not considered clinically significant AVN. Type 2 AVN is mainly manifested as lateral physical damage. In this study, we defined type 2, type 3 and type 4 AVN as the clinically significant AVN. We also defined type 1 and type 2 AVN as the low-grade AVN group and type 3 and type 4 AVN as the high-grade AVN or severe group.

### Statistical analysis

Descriptive summary statistics (mean and standard deviation or median and range as appropriate) were calculated. A t test was used for continuous variables between the two groups. The chi-square test and Spearman’s rank correlation test were used to evaluate correlations between variables. The level of significance was set at *P* < 0.05. Statistical analysis was performed using SPSS 21.0 for Windows (IBM Corp., Armonk, New York).

## Results

### Sample characteristics

There were 101 children (119 hips) with DDH who met the inclusion criteria, of whom 12 were boys and 89 were girls. The left side was involved in 49 hips and the right in 34, and 18 cases were bilateral. There were 66 hips in Group A and 53 hips in Group B. The mean surgical age at open reduction was 17.0 months (12–24) with a mean last follow-up at 104.9 months (60.4–131.3). There was no statistical difference of surgical age between the two groups (17.2 vs. 16.4 months). Based on preoperative IHDI grade, 10 hips were grade 2, 67 hips were grade 3, and 42 hips were grade 4. The mean AI of the affected hip preoperatively was 40.3 ± 5.3 degrees (Table 1).

**Table 1:** Baseline characteristics of the children in the study

Characteristic	Total *n* = 101 (hips, *n* = 119)
Gender (male/female)	12/89
Side (left/right/bilateral)	49/34/18
Preoperative IHDI grade (II/III/IV)	10/67/42
Preoperative AI (degree)	40.3
Mean age at surgery (months)	17
All types of AVN (Group A/B)	21/18
Significant AVN (Group A/B)	18/10
Severe AVN (Group A/B)	12/3
Mean follow-up time (months)	104.9

*IHDI* The International Hip Dysplasia Institute classification, *AI* acetabular index, *AVN* avascular necrosis

Further surgery was necessary in 36 hips. Fifteen hips underwent a pelvic osteotomy: 12 Salter and 3 Pemberton. Twenty-one hips underwent a pelvic osteotomy combined with a derotation/varus proximal femoral osteotomy, and 14 Salter, six Pemberton, and one Dega pelvic osteotomies were carried out.

### Complications

There were no other serious complications, such as redislocation or fracture, in either group. Superficial wound infection was seen in three patients.

### Avascular necrosis

All the hips were assessed using the Kalamchi and MacEwen classification. Of the 119 hips treated, type-1 temporary AVN was found in 11 hips, specifically three in group A and eight in group B. The rate of clinically significant AVN (types 2 to 4) was 23.5%; specifically, there was a total of 10 hips in Group B (18.9%) and 18 in Group A (27.3%), while there was severe AVN (types 3 and 4) in three hips in Group B and 13 in Group A (Table 1). There was no statistical difference in total AVN or clinically significant AVN incidence between the two groups (*P* = 0.804 and *P* = 0.283). However, a higher rate of severe AVN was identified in Group A, which consisted of those with rectus-sparing in the surgical procedure (19.7% vs. 5.7%, *P* = 0.026).

### Operation time and bleeding volume

The mean operation time on the unilateral hip joint in group A and group B was 30.1 min and 29.8 min, respectively, and there was no significant difference. The mean intraoperative bleeding volume of the unilateral hip joint in group A and group B was 12.4 ml and 11.6 ml, respectively, and there was no significant difference between the two groups.

### The presence of an ossific nucleus, IHDI classification, age and abducted angle

There was no significant correlation between the postoperative AVN degree and the preoperative IHDI classification grade (*P* = 0.117), preoperative AI (*P* = 0.436), presence of an ossific nucleus (*P* = 0.141), or abducted angle (*P* = 0.327). The average abducted angle was 61.5 ± 7.2 degrees. However, the postoperative AVN degree was lower among infants of a younger age undergoing surgery (*P* = 0.002).

## Discussion

The most important finding of this study was that preserving the rectus femoris in the modified Smith–Petersen approach could reduce severe AVN incidence in open reduction for DDH of infants between 12 and 24 months of age. Within this time spectrum, earlier open reduction can reduce postoperative AVN.

AVN is the most important complication after DDH treatment. It can result in proximal femoral growth disturbances leading to hip dysfunction, pain, and, eventually, osteoarthritis [12]. How to protect blood vessels in open reduction and reduce the incidence and degree of AVN of the femoral head remain pressing concerns for pediatric orthopedic scholars.

Previous studies of the effect of previous hip surgery on the vascularization of the proximal femur have mainly focused on femoral neck fracture, surgical hip dislocation procedure, and antegrade intramedullary femoral nailing, but there is little research about pediatric DDH in relation to this issue [13]. Christoph Hartog et al. found that arterial perfusion of the proximal femur is easily impaired after adult hip surgery, particularly after an anterior approach [14]. The incidence of AVN after open reduction for DDH varies between 4 and 66% [15]. The rate of clinically significant AVN in this study was 23.5%; however, the probability of occurrence was significantly lower in group B. Jia et al. also reported that the probability of AVN in the Smith–Petersen approach, preserving the rectus femoris muscle, was 8%, which was significantly lower than had been previously reported [16]. Similarly, the incidence of clinically significant and severe AVN in the modified Smith–Petersen approach with rectus-sparing (Group B) was 18.9% and 5.7%, respectively, in this study.

In the literature reviewed, barely any research reports on AVN incidence in the modified Smith–Petersen approach, preserving the rectus femoris muscle, versus the classical Smith–Petersen approach for open reduction in DDH during walking age. The reason for the lower AVN incidence in group B may be that it not only preserves the anatomical structure of the rectus femoris, but also requires less vessel dissection in this surgical technique and protects the blood supply of the femoral head. In most adult cases, the retinacular system via the medial femoral circumflex artery provides the dominant blood supply to the femoral head, and sometimes, the inferior gluteal artery provides the dominant supply [17].

However, the blood supply of the femoral head at all ages shows obvious evolution in the process from birth to adulthood. The arterial supply for the proximal femur in children consists of three parts: an extracapsular arterial ring, intracapsular ascending cervical arteries, and intracapsular subsynovial ring. The extracapsular arterial ring is at the femoral neck base and is formed by the junction of branches from the lateral and medial circumflex arteries [18, 19]. The lateral femoral circumflex artery makes a blood contribution of approximately 48% to the anteroinferior femoral neck [20]. The complex anatomical relationships of these arteries to the musculature surrounding the hip joint make them susceptible to injury during hip surgery [13]. The direct head of the rectus tendon retained in group B of this study has a broad insertion on the anterior inferior iliac spine [21, 22]. The anterior inferior iliac spine is adjacent to the lateral circumflex femoral artery and femoral nerve. Posterior to the rectus femoris, the lateral femoral circumflex artery branches into ascending, transverse, and descending branches. The ascending branch of the lateral femoral circumflex artery supplies the hip capsule and also gives rise to the anterior retinacular artery, which enters the hip capsule and supplies the femoral neck [23]. The femoral artery supplies the quadriceps muscle, and the ascending branch of the lateral femoral circumflex artery specifically nourishes the rectus femoris [23]. Besides, the direct head of the rectus tendon and the proximal end of the rectus femoris are close to the upper edge of the acetabulum and the anterior articular capsule, which could be supplied by a blood vessel demonstrating a capsular branch and an anastomosis between the supra-acetabular branch of the superior gluteal artery and the ascending branch of the lateral femoral circumflex artery [17]. Therefore, rectus-sparing and the less dissection associated with this surgical technique together in the proximal region of the rectus femoris in open reduction can reduce the iatrogenic decline of femoral head blood supply.

Most studies of femoral head blood supply have been based on healthy adults with a normal femoral head/acetabular relationship, unlike in the present study. The first point is that the femoral head moves significantly in the direction of the upper and outer acetabulum in DDH. The second point is that the subjects in this study are children aged just one to two years. In patients with Paley type 1 or 2 congenital femoral deficiency with changed proximal femur morphology, the femoral neurovascular bundle seems to be pulled by the abnormal proximal femur and closer to the anterior inferior iliac spine, which is the insertion of the direct head of the rectus tendon. The distance from the femoral neurovascular bundle to the anterior inferior iliac spine was significantly different to the unaffected side (13 mm vs. 23 mm) [24]. In this study, the affected hips have obvious morphological abnormalities of the proximal femur and move to the upper and outer direction of the acetabulum. It can be speculated that the origin of the lateral femoral circumflex artery and medial femoral circumflex artery located in the inner and lower part of the femoral head will be closer to the anterior inferior iliac spine than the normal healthy side, so injury may be more likely in the open reduction in the anterior approach. In addition to the relative relationship between the femoral head and the acetabulum, other factors may affect the distance between the neurovascular bundle and hip joint. Mehta et al. found that patients with a low body weight may be at a higher risk of iatrogenic injury due to the reduced distance of the neurovascular structures to the hip [25]. The children in this study are only one to two years old and thus obviously weigh less than adults, and this will cause the neurovascular bundle to be closer to the hip capsule. Considering that abnormal morphology of the femoral head will lead to arterial traction, it is very important to protect the arterial blood supply of the hip joint in open reduction.

The effect of operative age on femoral head necrosis is complex and controversial. Novais et al. found that age at reduction (less than 12 months vs. more than 12 months) was not associated with AVN after open reduction. Gardner et al. found that a higher rate of AVN was identified when surgery was performed in children aged less than 12 months following medial open reduction [4]. We speculate that the younger the age, the smaller the distance between the hip joint capsule and blood vessels. Considering the medial circumflex femoral artery traverses the anteromedial capsule in the medial approach, we speculate that, if the child is younger, the probability of AVN will be higher following medial open reduction. Cholawish et al. found that at the anterior portal in fresh infantile cadavers, the transverse branch of the lateral femoral circumflex artery only had an average distance of 8.5 mm, and the most lateral branch of the lateral femoral cutaneous nerve was frequently injured [26]. In contrast, Edward reported that in an adult cadaver study, the average distance between the femoral artery and the anterior hip capsule was 21 mm [27]. However, older age when receiving open reduction will also be accompanied by longer walking time, and the stress state of the hip joint during walking will obviously lead to the deterioration of and pathological changes to the hip joint. This pathological aggravation includes the upward and outward displacement of the femoral head, lengthening of the joint capsule, and narrowing of the anterior joint capsule. These changes caused by walking may alter the relationship between the blood vessels in the proximal femoral head and anterior articular capsule.

This study has several limitations. Firstly, it is a retrospective study, which makes it impossible to avoid selection bias. Secondly, this study did not evaluate hip muscle strength after rectus femoris preservation, and additional investigation is needed to determine whether the rectus-preserving approach allows for improvement in functional recovery, including hip flexion strength. Thirdly, the study did not directly measure the distance between the femoral artery and the anterior capsule or anterior inferior iliac spine or evaluate the relationship between this distance and the degree of hip dislocation. Fourthly, this study just included mid-term data and needs further observation. Five-year follow-up is not sufficient to provide true finial incidence of type 2 AVN because many will appear in final two years prior to physeal closure.

## Conclusion

The most important finding of this study was that the modified Smith–Petersen approach, which preserves the rectus femoris, could reduce severe AVN relative to the classical Smith-Petersen approach in open reduction for DDH at walking age basing on mid-term data. We also found that earlier open reduction for hip dislocation can reduce the degree of postoperative AVN at walking age.

## Data Availability

The datasets are available from the corresponding author on reasonable request.

## Abbreviations

DDH: Developmental dysplasia of the hip
IHDI: The International Hip Dysplasia Institute classification
AI: Acetabular index
AVN: Avascular necrosis
